# Adherence to dietary guidelines and associated factors among rural Australian cancer survivors: a cross-sectional study

**DOI:** 10.1007/s00520-024-08494-3

**Published:** 2024-05-03

**Authors:** Michael James Leach, Georgina Barber, Stephanie Monacella, Philip Jamieson, Thi Trinh, Ngan Vo, Ulla Schmidt, Anny Byrne, Eli Ristevski

**Affiliations:** 1 School of Rural Health, Monash University, Bendigo, VIC Australia; 2 West Gippsland Healthcare Group, Warragul, VIC Australia; 3 School of Rural Health, Monash University, Warragul, VIC Australia

**Keywords:** Dietetics, Nutrition, Supportive care, Rural, Cancer, Survivorship

## Abstract

**Purpose:**

This study aimed to explore levels of adherence to dietary guidelines, and factors associated with dietary guideline adherence, among rural Australian cancer survivors.

**Methods:**

A cross-sectional study was undertaken. We recruited a convenience sample of adults with cancer who attended the chemotherapy day unit or allied health appointments at a rural hospital in Baw Baw Shire, Victoria, Australia, between August 2017 and December 2021. Dietary guideline adherence was assessed by cross-referencing participants’ responses to an adapted version of the Dietary Questionnaire for Epidemiological Studies with dietary recommendations in Australian dietary guidelines. Binary logistic regression was used to assess factors associated with dietary guideline adherence for fruits and whole red meats.

**Results:**

There were 107 rural cancer survivors (median age, 67 years). Dietary guideline adherence was highest for alcohol (88%) followed by whole red meats (63%), fruits (56%), processed red meats (24%), cereals/breads/grains (7%), and vegetables (4%). Relative to those aged < 65 years, 65–74-year-olds had 5.7-fold greater odds (adjusted odds ratio (aOR) = 5.74, 95% confidence interval (CI) = 1.91–17.17) of adhering to the dietary guideline for fruits. Relative to those who had completed/ceased treatment, participants who were currently receiving treatment had 78% lower odds (aOR = 0.22, 95% CI = 0.09–0.59) of adhering to the dietary guideline for fruits.

**Conclusion:**

This study contributes preliminary data on adherence to dietary guidelines and associated factors among rural Australian cancer survivors. Dietary guideline adherence varied across food groups and was mostly low, albeit not markedly worse than Australia’s national population for the fruits and vegetables groups. The mostly low adherence in our sample suggests a potential need to increase provision of dietary information, supportive care screening, and, wherever necessary, dietetics referrals, assessments, and interventions among rural cancer survivors. Larger, longitudinal studies of adherence to dietary guidelines and/or tailored, cancer-specific dietary recommendations should be undertaken in future.

## Introduction

Diet quality is an important consideration among cancer survivors [1, 2]. A higher quality diet is associated with improved survival and better quality of life in people who have been diagnosed with a range of tumour types, including breast, prostate, and colorectal cancers [1, 2]. Adherence to dietary guidelines varies considerably across cancer populations around the world. For instance, internationally, adherence to guidelines for both fruits and vegetables among cancer survivors has been found to vary from 9% in the Netherlands to 83% in the United States (US) [3]. Among Australian cancer survivors, adherence to guidelines for fruits has been found to be 58% and adherence to guidelines for vegetables 37% [4].

Both patients and health practitioners have roles to play in improving diet quality during cancer survivorship. Cancer survivors are more likely to make positive dietary changes or eat more fruits and vegetables when they do so with intent, motivation, and self-efficacy [5, 6]. However, known health system barriers prevent people with cancer from obtaining the support they need to improve their adherence to dietary guidelines. Cancer survivors have reported that, when seeking to improve their eating behaviours, they are impeded by non-specific dietary advice from healthcare workers as well as inadequate dietary follow-up and a lack of referrals to dietetics services [7]. These barriers to receiving dietary support are more pronounced in non-metropolitan (henceforth termed ‘rural’) areas than metropolitan areas [8].

Screening for dietary issues among people with cancer forms part of a broader supportive care screening process. In the state of Victoria, Australia during 2021, 36% of a representative sample of newly diagnosed cancer patients were screened for supportive care issues—well below the Victorian Government Department of Health’s target of 80% [9]. There is evidence to suggest that, in Victoria, 22% of referrals triggered by supportive care screening are referrals to dietetics services—the second most common referral type after social work (24%) [10]. Knowledge of demographic, medical, and lifestyle-related characteristics associated with adherence to dietary guidelines among rural cancer survivors could help identify priority groups for dietary screening, referral, assessment, and/or intervention, thereby informing the development of interdisciplinary models of supportive care involving dieticians and other health professionals—models of care aimed at improving the quality of dietary and other support services available to rural people with cancer. Few studies, however, have reported on adherence to dietary guidelines and/or associated factors among rural cancer survivors. In Australia, for example, two studies are known to have examined rural cancer survivors’ adherence to dietary guidelines [11, 12]. In an Australian cross-sectional study conducted among 63 cancer survivors in the Northern Territory (NT), the percentages of participants meeting dietary guidelines were 76% for whole red meat, 75% for alcohol, 73% for fruits, 33% for processed meats, and 10% for vegetables [11]. This NT study reported a higher percentage meeting dietary guidelines for fruits and lower percentages meeting dietary guidelines for meats among 20 Indigenous participants (fruits, 80%; whole red meats, 65%; processed meats, 20%) than 43 non-Indigenous participants (fruits, 70%; whole red meats, 81%; processed meats, 40%), although the statistical significance of associations between participant characteristics and meeting dietary guidelines was not assessed in the study due to its small sample size [11]. In an analysis of retrospective data from the South Australian Monitoring and Surveillance System, 46% and 15% of 916 rural cancer survivors met dietary guidelines for fruits and vegetables, respectively [12]. Associations between participant characteristics and meeting dietary guidelines were not assessed within the rural cancer survivors who participated in this South Australian study.

Due to these gaps in the literature, the present study was undertaken to explore levels of adherence to dietary guidelines and associated factors among rural Australian cancers survivors. We sought to answer two research questions: (1) What percentages of rural Australian cancer survivors meet dietary guidelines for specific food groups, such as fruits and whole red meats? (2) What demographic, medical, and lifestyle-related characteristics are associated with meeting dietary guidelines for specific food groups, such as fruits and whole red meats, among rural Australian cancer survivors?

## Methods

### Setting

The setting for this study has been described previously [13, 14]. Briefly, the study was set in Baw Baw Shire in rural Victoria, Australia. Rates of potentially modifiable risk factors, such as inadequate fruit intake, are higher at the population level in Baw Baw Shire than in the whole of the state of Victoria or Australia-wide [15]. Baw Baw Shire has one hospital with a chemotherapy day unit (CDU), where chemotherapy is offered 1 day per week. This CDU has the capacity to treat an average of 18 people with cancer per week.

### Design and sampling

The study design and sampling have been described and justified in detail previously [13]. Briefly, a cross-sectional study was undertaken using a set of baseline (pre-intervention) data collected for a prospective cohort study of a health coaching intervention (called the I.CAN Program) [14]. The study population comprised a convenience sample of people with cancer who, upon attending the CDU or allied health appointments at the rural hospital between August 2017 and December 2021, took up the offer to take part in the I.CAN Program. The inclusion criteria were age 18 years or older and any cancer diagnosis while the exclusion criteria were acute malnutrition and end-of-life care. There was no inclusion criterion for treatment completion because, in line with the National Coalition for Cancer Survivorship’s definition of survivorship [16], cancer survivors were broadly defined as those with a diagnosis of cancer rather than those who had completed treatment.

### Data collection

All data were collected on paper-based forms, either directly from participants or from hospital medical records, ahead of entry into a secure, password-protected Microsoft Access database (Microsoft Corp., Redmond, WA, USA).

### Measures of adherences to dietary guidelines

Adherence to dietary guidelines was measured using baseline data collected via an adapted version of the Dietary Questionnaire for Epidemiological Studies (Version 2), which was originally developed by Giles and Ireland [17] and validated for use in Australian adults [18]. The hospital-based dietetics team adapted the original version of this questionnaire to make it shorter (two A4 pages) and simpler to complete, while still covering the same content and using the same scale. Our adapted version of this questionnaire, called the I.CAN Food Frequency Questionnaire (FFQ), had been successfully used in clinical practice among rural people with lower literacy levels prior to the present study. The I.CAN FFQ asks respondents to indicate the typical frequency with which they consume particular food types. The frequency of food consumption is measured on an 8-point scale: rarely or never (response option 1), less than 1 serve a week (response option 2), 1 serve a week (response option 3), 2–3 serves a week (response option 4), 4–6 serves a week (response option 5), 1–2 serves a day (response option 6), 3–4 serves a day (response option 7), and 5 + serves a day (response option 8).

In the present study, relevant individual food types within the following six food groups were assessed due to their importance to cancer [19] and/or their inclusion in previous studies conducted among cancer survivors [3–5, 11, 12]: fruits, vegetables, cereals/breads/grains, whole red meats, processed red meats, and alcohol. For each individual food type (e.g., salad, vegetables (except potatoes) and beans/pulses for vegetables) in the I.CAN FFQ, adherence to dietary guidelines was determined using recommendations published in the Australian Dietary Guidelines [20] or the Australian Guidelines to Reduce Health Risks from Drinking Alcohol [21]. Table 1 shows the data rules used to determine each participant’s adherence to dietary guidelines for each individual food type across the six food groups assessed. While the Australian Dietary Guidelines recommend at least 2 serves of fruit and no more than 1 serve of whole red meat per day 1–4 serves of fruit per day and up to 6 serves of whole red meat per week denoted adherence for these food groups in the present study. This is because our I.CAN FFQ tool reused validated Dietary Questionnaire for Epidemiological Studies (Version 2) [17, 18] response options, which were developed for consistent use across different food groups and which do not precisely match recommendations for all food groups in the Australian Dietary Guidelines [20]. For each food group, a binary dietary guideline adherence variable with categories of ‘adherence’ (the non-reference group) and ‘non-adherence’ (the reference group) was created. As the food groups ‘vegetables’ and ‘cereals/breads/grains’ comprised multiple individual food types (Table 1), dietary guideline adherence for these two food groups was defined as meeting the Australian Dietary Guidelines [20] for one or more individual food types. Total alcohol intake was not directly measured in the present study; intake of specific types of alcohol was measured instead. As there were multiple alcohol categories and all alcohol types are considered harmful, alcohol guideline adherence was defined as meeting the Australian Guidelines to Reduce Health Risks from Drinking Alcohol [21] for all individual alcohol types.

**Table 1:** Data rules used to determine whether rural Australian cancer survivors adhered to Australian dietary guidelines for individual food types across six food groups

Food group	Individual food type(s)	Data rule for guideline adherence
Fruits	Tinned/fresh fruits	1–2 serves a day (response option 6) or 3–4 serves a day (response option 7)
Vegetables	Salad	5 + serves a day (response option 8)
	Vegetables^**†**^	5 + serves a day (response option 8)
	Beans/pulses	5 + serves a day (response option 8)
Cereals/breads/grains	High-fibre cereals	3–4 serves a day (response option 7) or 5 + serves a day (response option 8)
	Wholegrain/multigrain breads	3–4 serves a day (response option 7) or 5 + serves a day (response option 8)
	Rice/pasta	3–4 serves a day (response option 7) or 5 + serves a day (response option 8)
Whole red meat	Beef, lamb, pork, ham^‡^	2–3 serves a week (response option 4) or 4–6 serves a week (response option 5)
Processed red meats	Sausages, bacon, corned beef, meat pies/pasties, burgers	Rarely or never (response option 1) only
Alcohol	Beer (low alcohol)	Rarely or never (response option 1), less than 1 serve a week (response option 2), 1 serve a week (response option 3), or 2–3 serves a week (response option 4)
	Beer (full strength)	Rarely or never (response option 1), less than 1 serve a week (response option 2), 1 serve a week (response option 3), or 2–3 serves a week (response option 4)
	Wine (red/white)	Rarely or never (response option 1), less than 1 serve a week (response option 2), 1 serve a week (response option 3), or 2–3 serves a week (response option 4)
	Fortified wines/port/sherry	Rarely or never (response option 1), less than 1 serve a week (response option 2), 1 serve a week (response option 3), or 2–3 serves a week (response option 4)
	Spirits/liqueurs	Rarely or never (response option 1), less than 1 serve a week (response option 2), 1 serve a week (response option 3), or 2–3 serves a week (response option 4)

^**†**^Excluding potatoes

^‡^Including steaks, roasts, mince, or chops

### Measures of demographic, medical, and lifestyle-related characteristics

Age at baseline was defined as a continuous variable as well as a categorical variable with categories of < 65, 65–74, and ≥ 75 years. In terms of further demographic characteristics, binary variables were created for gender (male or female) as well as self-identified Aboriginal and/or Torres Strait Islander origin (yes or no) and born outside Australia (yes or no). As nearly half of participants had breast cancer, cancer type was defined as a binary variable with categories of ‘breast’ and ‘other’. As the survivorship phase of the cancer journey may be subdivided into the time before and after completion or cessation of treatment, treatment status was defined as a binary variable with categories of ‘current’ and ‘completed or ceased’. Two lifestyle-related characteristics, physical activity and obesity, were also assessed. Physical activity was measured via a validated instrument: the Godin-Shephard Leisure-Time Physical Activity Questionnaire [22, 23]. Physical activity scores were collapsed into a binary variable with categories of ‘sufficiently active’ (scores ≥ 14) and ‘insufficiently active’ (scores < 14) [24]. Obesity was assessed by, firstly, calculating body mass index (BMI) in kilogrammes (kg) of weight per metre-squared (m^2^) of height, and, secondly, creating a binary obesity variable with the following categories: ‘yes’ (BMI ≥ 30.0 kg/m^2^) and ‘no’ (BMI < 30.0 kg/m^2^) [25].

### Statistical analysis

Age in years was described using the median (interquartile range (IQR)) while all categorical, including binary, variables were described by calculating the frequency and percentage. Cross-tabulations were produced for two-way combinations of each binary dietary guideline adherence variable with each of the categorical, including binary, demographic, medical, and lifestyle-related, characteristic variables. If the dietary guideline adherence variable for a particular food group had expected (as opposed to observed) cell counts ≥ 5 in at least 80% of each cross-tabulation’s cells across all cross-tabulations, then that dietary guideline adherence variable was treated as a dependent variable for the purposes of regression analysis. Due to low expected cell counts in cross-tabulations (data not shown), binary logistic regression analyses were not undertaken for the following four dependent variables: adherence to dietary guidelines for each of vegetables, cereals/breads/grains, processed red meats, and alcohol. Due to adequate expected cell counts in cross-tabulations (data not shown), binary logistic regression analyses were undertaken for two dependent variables: adherence to dietary guidelines for each of fruits and whole red meats. For each of these two food groups separately, univariable and multivariable binary logistic regression models were used to assess associations between each categorical/binary demographic/medical/lifestyle-related characteristic variable (i.e. the independent variables) and the given binary dietary guideline adherence variable (i.e. the dependent variable). This involved the estimation of odds ratios (ORs) and 95% confidence intervals (CIs) in univariable models as well as the estimation of adjusted odds ratios (aORs) and 95% CIs in multivariable models. If the 95% CI around a given OR or aOR excluded the null value of 1.00, then the effect was deemed to be statistically significant at the 5% level and there was, thus, said to be an association between the relevant independent and dependent variables. Missing data on dependent and independent variables were handled through a complete-case approach, which involves excluding participants with missing data on one or more variables from the sample and analysis. All statistical analyses were performed in Stata v15.0 (StataCorp, College Station, TX, USA).

### Ethical considerations

Ethical approval to undertake this study was obtained from West Gippsland Healthcare Group Human Research Ethics Committee (ID: ICAN), Latrobe Regional Hospital Human Research Ethics Committee (ID: 2020–14), and Monash University Human Research Ethics Committee (ID: 11890). This study was conducted in accordance with the Declaration of Helsinki (2013) and the National Statement on Ethical Conduct in Human Research 2007 (Updated 2018). Before providing written informed consent, all participants were informed about the study both verbally and in writing.

## Results

### Sample

Overall, 112 rural cancer survivors met the eligibility criteria. Of them, 107 participants (96%) had complete data for all variables and, in line with the complete-case approach, were included in the analysis. Among the five excluded participants, one did not respond to the I.CAN FFQ, three had missing obesity data due to a lack of available information on weight and/or height, and one had missing data on both the I.CAN FFQ and obesity. Descriptive statistics on all demographic, medical, and lifestyle-related characteristics are shown in Table 2. The median (IQR) age of included participants was 67 (19) years, 72% were female, and 86% were born in Australia (Table 2). None of the 107 included participants self-identified as Aboriginal and/or Torres Strait Islander. Nearly half of participants had breast cancer (46%) and were currently receiving treatment (49%). In terms of lifestyle-related characteristics, 36% had sufficiently physical activity and 41% presented with obesity.

**Table 2:** Rural Australian cancer survivors’ demographic, medical, and lifestyle-related characteristics, overall in the whole sample and in cross-tabulations with adherence to Australian dietary guidelines for six food groups (*N* = 107)

	Overall	Adherence to dietary guidelines, *n* (row %)^†^											
Characteristic	*n* (col. %)^†^	Fruits		Vegetables		Cereals/breads/grains		Whole red meats		Processed red meats		Alcohol	
Category^†^		Yes	No	Yes	No	Yes	No	Yes	No	Yes	No	Yes	No
Demographic													
Age in years (median, IQR)	67.0 (18.5)	71.0 (14.9)	62.0 (21.7)	52.8 (12.9)	68.4 (18.3)	69.1 (21.2)	67.0 (18.3)	68.4 (16.0)	64.7 (22.2)	64.2 (21.8)	68.4 (16.6)	66.0 (18.2)	73.9 (14.4)
Age group													
< 65 years	49 (45.8)	21 (42.9)	28 (57.1)	4 (8.2)	45 (91.8)	4 (8.2)	45 (91.8)	29 (59.2)	20 (40.8)	14 (28.6)	35 (71.4)	45 (91.8)	4 (8.2)
65–74 years	41 (38.3)	28 (68.3)	13 (31.7)	0 (0.0)	41 (100.0)	2 (4.9)	39 (95.1)	26 (63.4)	15 (36.6)	9 (22.0)	32 (78.0)	37 (90.2)	4 (9.8)
≥ 75 years	17 (15.9)	11 (64.7)	6 (35.3)	0 (0.0)	17 (100.0)	2 (11.8)	15 (88.2)	12 (70.6)	5 (29.4)	3 (17.6)	14 (82.4)	12 (70.6)	5 (29.4)
Gender													
Female	77 (72.0)	46 (59.7)	31 (40.3)	4 (5.2)	73 (94.8)	4 (5.2)	73 (94.8)	47 (61.0)	30 (39.0)	24 (31.2)	53 (68.8)	69 (89.6)	8 (10.4)
Male	30 (28.0)	14 (46.7)	16 (53.3)	0 (0.0)	30 (100.0)	4 (13.3)	26 (86.7)	20 (66.7)	10 (33.3)	2 (6.7)	28 (93.3)	25 (83.3)	5 (16.7)
Country of birth													
Australia	92 (86.0)	48 (52.2)	44 (47.8)	3 (3.3)	89 (96.7)	6 (6.5)	86 (93.5)	59 (64.1)	33 (35.9)	19 (20.7)	73 (79.3)	80 (87.0)	12 (13.0)
Country other than Australia	15 (14.0)	12 (80.0)	3 (20.0)	1 (6.7)	14 (93.3)	2 (13.3)	13 (86.7)	8 (53.3)	7 (46.7)	7 (46.7)	8 (53.3)	14 (93.3)	1 (6.7)
Medical													
Cancer type													
Other^‡^	58 (54.2)	33 (56.9)	25 (43.1)	1 (1.7)	57 (98.3)	5 (8.6)	53 (91.4)	37 (63.8)	21 (36.2)	12 (20.7)	46 (79.3)	51 (87.9)	7 (12.1)
Breast	49 (45.8)	27 (55.1)	22 (44.9)	3 (6.1)	46 (93.9)	3 (6.1)	46 (93.9)	30 (61.2)	19 (38.8)	14 (28.6)	35 (71.4)	43 (87.8)	6 (12.2)
Currently receiving treatment													
No (completed or ceased treatment)	55 (51.4)	38 (69.1)	17 (30.9)	53 (96.4)	2 (3.6)	4 (7.3)	51 (92.7)	36 (65.5)	19 (34.5)	12 (21.8)	43 (78.2)	45 (81.8)	10 (18.2)
Yes (current treatment)	52 (48.6)	22 (42.3)	30 (57.7)	50 (96.2)	2 (3.8)	4 (7.7)	48 (92.3)	31 (59.6)	21 (40.4)	14 (26.9)	38 (73.1)	49 (94.2)	3 (5.8)
Lifestyle-related													
Sufficient physical activity^§^													
No	69 (64.5)	35 (50.7)	34 (49.3)	1 (1.4)	68 (98.6)	5 (7.2)	64 (92.8)	44 (63.8)	25 (36.2)	11 (15.9)	58 (84.1)	63 (91.3)	6 (8.7)
Yes	38 (35.5)	25 (65.8)	13 (34.2)	3 (7.9)	35 (92.1)	3 (7.9)	35 (92.1)	23 (60.5)	15 (39.5)	15 (39.5)	23 (60.5)	31 (81.6)	7 (18.4)
Obesity^¶^													
No (BMI < 30 kg/m^2^)	63 (58.9)	40 (63.5)	23 (36.5)	2 (3.2)	61 (96.8)	6 (9.5)	57 (90.5)	39 (61.9)	24 (38.1)	20 (31.7)	43 (68.3)	54 (85.7)	9 (14.3)
Yes (BMI ≥ 30 kg/m^2^)	44 (41.1)	20 (45.4)	24 (54.5)	2 (4.5)	42 (95.5)	2 (4.5)	42 (95.5)	28 (63.6)	16 (36.4)	6 (13.6)	38 (86.4)	40 (90.9)	4 (9.1)

*n*, frequency; *OR*, odds ratio (in univariable binary logistic regression model); *IQR*, interquartile range; *BMI*, body mass index; *kg*, kilogrammes; *m*, metres

^**†**^Unless otherwise stated

^‡^ ‘Other’ (i.e. non-breast) cancers include central nervous system, colorectal, genitourinary, gynaecological, haematological, lung, and upper gastrointestinal cancers

^§^Classified as a score ≥ 14 on the Godin-Shephard physical activity scale[22, 23]

^¶^Classified using the World Health Organization’s definition[25]

### Adherence to dietary guidelines

Among the 107 participants, adherence to dietary guidelines was highest for alcohol (88%) followed by whole red meats (63%) and fruits (56%), and relatively low for processed red meats (24%), cereals/breads/grains (7%), and vegetables (4%) (Table 3).

**Table 3:** Frequency and percentage of rural Australian cancer survivors who adhered to Australian dietary guidelines for fruits, vegetables, cereals, whole red meats, processed red meats, and alcohol (*N* = 107)

Food group	Individual food type(s) included	Data rule for guideline adherence	*n* (row %)	
			Adherence	Non-adherence
Fruits	Tinned/fresh fruit	1–2 serves a day (response option 6) or 3–4 serves a day (response option 7)	60 (56.1)	47 (43.9)
Vegetables	Salad, vegetables^†^, beans/pulses	5 + serves a day (response option 8)	4 (3.7)	103 (96.3)
Cereals/breads/grains	High-fibre breakfast cereal, multigrain/wholemeal bread, rice/pasta	3–4 serves a day (response option 7) or 5 + serves a day (response option 8)	8 (7.5)	99 (92.5)
Whole red meats	Beef, lamb, pork, ham^‡^	2–3 serves a week (response option 4) or 4–6 serves a week (response option 5)	67 (62.6)	40 (37.4)
Processed red meats	Sausages, bacon, corned beef, meat pies/pasties, burgers	Rarely or never (response option 1) only	26 (24.3)	81 (75.7)
Alcohol	Beer (low alcohol), beer (full strength), wine (red/white), fortified wines/port/sherry, spirits/liqueurs	Rarely or never (response option 1), less than 1 serve a week (response option 2), 1 serve a week (response option 3), or 2–3 serves a week (response option 4)	94 (87.9)	13 (12.1)

*n*, frequency

^**†**^Excluding potatoes

^‡^Including steaks, roasts, mince, or chops

### Correlates of adherence to dietary guidelines

Table 2 shows observed cell counts for all cross-tabulations of participants’ demographic, clinical, and lifestyle-related characteristics by their adherence to dietary guidelines for the six different food groups. Table 4 shows univariable and multivariable binary logistic regression results for associations between participant characteristics and adherence to dietary guidelines for each of fruits and whole red meats. After adjusting for all other independent variables assessed, age 65–74 years was significantly positively associated with meeting the fruit guideline while currently receiving treatment was significantly negatively associated with meeting the fruit guideline. Compared with participants aged less than 65 years, participants aged 65–74 years had 5.7-fold greater odds (aOR = 5.74, 95% CI = 1.91–17.17) of meeting the dietary guideline for fruits. Conversely, compared with participants who had completed or ceased treatment, participants who were currently receiving treatment had 78% lower odds (aOR = 0.22, 95% CI = 0.09–0.59) of meeting the dietary guideline for fruits. No demographic, medical, or lifestyle-related factors were significantly associated with adherence to the dietary guideline for whole red meats.

**Table 4:** Associations between rural Australian cancer survivors’ demographic, medical, and lifestyle-related characteristics and their adherence to Australian dietary guidelines for fruits and whole red meats (*N* = 107)

Characteristic	Adherence to dietary guidelines for fruits		Adherence to dietary guidelines for whole red meats	
Category	OR (95% CI)	aOR (95% CI)	OR (95% CI)	aOR (95% CI)
Demographic				
Age group				
< 65 years	1.00	1.00	1.00	1.00
65–74 years	2.87 (1.21–6.84)*	5.74 (1.91–17.17)*	1.20 (0.51–2.81)	1.28 (0.52–3.16)
≥ 75 years	2.44 (0.78–7.68)	2.14 (0.51–8.92)	1.66 (0.50–5.43)	1.78 (0.47–6.72)
Gender				
Female	1.00	1.00	1.00	1.00
Male	0.59 (0.25–1.38)	0.32 (0.10–1.09)	1.28 (0.53–3.10)	1.19 (0.39–3.58)
Country of birth				
Australia	1.00	1.00	1.00	1.00
Country other than Australia	3.67 (0.97–13.9)	2.82 (0.62–12.9)	0.64 (0.21–1.92)	0.56 (0.17–1.82)
Medical				
Cancer type				
Other^†^	1.00	1.00	1.00	1.00
Breast	0.93 (0.43–2.00)	0.77 (0.26–2.28)	0.90 (0.41–1.97)	1.02 (0.39–2.69)
Currently receiving treatment				
No (completed or ceased treatment)	1.00	1.00	1.00	1.00
Yes (current treatment)	0.33 (0.15–0.73)*	0.22 (0.09–0.59)*	0.78 (0.36–1.71)	0.74 (0.32–1.69)
Lifestyle-related				
Sufficient physical activity^‡^				
No	1.00	1.00	1.00	1.00
Yes	1.87 (0.82–4.24)	1.35 (0.49–3.70)	0.87 (0.39–1.97)	0.88 (0.36–2.15)
Obesity^§^				
No (BMI < 30 kg/m^2^)	1.00	1.00	1.00	1.00
Yes (BMI ≥ 30 kg/m^2^)	0.48 (0.22–1.05)	0.56 (0.21–1.51)	1.08 (0.49–2.39)	1.04 (0.42–2.58)

*n*, frequency; *OR*, odds ratio (in univariable binary logistic regression model); *CI*, confidence interval; *aOR*, adjusted odds ratio (in multivariable binary logistic regression model); *IQR*, interquartile range; *BMI*, body mass index; *kg*, kilogrammes; *m*, metres

* Statistically significant at the 5% level

^**†**^ ‘Other’ (i.e. non-breast) cancers include central nervous system, colorectal, genitourinary, gynaecological, haematological, lung, and upper gastrointestinal cancers

^‡^Classified as a score ≥ 14 on the Godin-Shephard physical activity scale [22, 23]

^§^Classified using the World Health Organization’s definition [25]

## Discussion

In our study of adherence to dietary guidelines among 107 rural Australian cancer survivors, 88% of participants adhered to the dietary guideline for alcohol, 63% adhered to the dietary guideline for whole red meats, and 56% adhered to the dietary guideline for fruits. Fewer than one-quarter of participants adhered to the dietary guidelines for each of processed red meats, cereals/breads/grains, and vegetables. These findings demonstrate room for improvement in rural cancer survivors’ eating habits across all food groups assessed.

The levels of adherence to dietary guidelines for alcohol, whole red meats, fruits, and vegetables in our study population may be compared with the corresponding levels found in other Australian populations. It is important to note, however, that definitions of dietary guideline adherence differed somewhat across studies. In our study, the observed level of alcohol guideline adherence (88%) was higher than the corresponding levels of alcohol guideline adherence among 63 rural cancer survivors from Australia’s NT (75%) [11] as well as among people with or without cancer Australia-wide (74%). [26]. The proportion of our study population who adhered to the dietary guideline for whole red meats (63%) was lower than the proportion of rural cancer survivors from the NT who adhered to this guideline (76%) [11]. The level of adherence to the dietary guideline for fruits in our study (56%) was higher than the corresponding proportions among 916 rural South Australian cancer survivors (46%) [12] as well as people with or without cancer Australia-wide (44%) [27], yet lower than the corresponding proportion in rural cancer survivors from the NT (73%) [11]. The level of adherence to the dietary guideline for vegetables in our study (3.7%) was lower than the corresponding proportions among rural cancer survivors in both South Australia (15%) [12] and the NT (10%) [11] as well as among people with or without cancer Australia-wide (6.5%) [27]. The fact that dietary guideline adherence in our sample of rural cancer survivors is 12 percentage points higher than Australia-wide for fruits and only 2.8 percentage points lower than Australia-wide for vegetables suggests that suboptimal dietary quality is not an issue specific to rural cancer survivors: it is an issue in the Australian population more broadly [27].

In terms of international benchmarking, the levels of adherence to the dietary guidelines in our study cannot be compared with international studies due to markedly different definitions of guideline adherence. For instance, the past study conducted among Dutch cancer survivors used a different definition of fruit guideline adherence (2 + serves per week) than our study (1–4 serves per day), as well as a different definition of vegetable guideline adherence (≥ 200 g of vegetables per week) compared with our study (5 + serves a day) [5].

In our study, age and treatment status were associated with adherence to dietary guidelines for fruit consumption. The odds of adherence to Australia’s dietary guideline for fruits were significantly greater among those aged 65–74 years, relative to those aged < 65 years, and significantly lower among those currently receiving treatment, relative to those who had completed or ceased treatment. The former finding may reflect the fact that health professionals such as nurses and geriatricians tend to prioritise nutritional assessments in elderly patients [28]; younger adults could be overlooked. The latter finding may be related to reduced appetite during cancer treatment [29], which is likely due to treatment side effects [30]. Reduced appetite during cancer treatment has previously been shown to occur in 40% of 1199 people receiving treatment for cancer in metropolitan US hospital settings [29].

As a high-quality diet is important for primary and secondary cancer prevention as well as overall health and well-being, dieticians are one of many health professionals who should be involved in cancer care [31]. Our study provides some preliminary evidence to suggest that rural cancer survivors aged < 65 years and on active treatment may be more suitable candidates to screen for a particular dietary issue: suboptimal fruit intake. However, most or even all cancer survivors should ideally be provided with dietary information, routinely screened for any dietary issues [9] and, if necessary, referred to a dietician. This could be achieved through broader information provision initiatives and supportive care screening processes, whereby cancer survivors are screened for any health problems at the time of cancer diagnosis as well as during and after active treatment. With regard to screening for dietary issues in particular, supportive care screening tools could incorporate nutritional assessment instruments [32], dietary questionnaires, or problem checklists mentioning appetite loss and other dietary issues. It is important to note, however, that person-centred barriers to identifying and managing dietary issues among cancer survivors (e.g. cost to the patient in terms of both time and money [8]) also need to be considered, particularly in rural settings such as the current study’s setting.

The results of the present study should not be interpreted without considering the study’s limitations. Firstly, tailored dietary recommendations for individual cancer survivors may differ from dietary guidelines developed for the general Australian population, which were used to define guideline adherence in our study. In particular, people with cancer typically need to increase their intake of protein and energy to prevent or treat malnutrition [33]. We partly addressed this limitation through the exclusion of participants who experienced acute malnutrition. Secondly, measures of adherence to dietary guidelines were likely affected by misclassification bias. For example, classifying those participants who had 1 serve of fruit per day as being guideline-adherent and classifying those participants who had 7 serves of whole red meat per week as being nonadherent may have led to misclassification and, thus, overestimated the percentage of participants meeting the fruit guideline and underestimated the percentage meeting the whole red meat guideline. Additionally, assessing alcohol intake across different alcohol types rather than total alcohol intake may have led to overestimation of the percentage of participants meeting the alcohol guideline. Thirdly, our use of a cross-sectional study design means that the two statistically significant associations discussed above are not necessarily causal relationships. Fourthly, the lack of adjustment for certain factors potentially related to both regression model covariates and the dietary guideline adherence outcomes (e.g. person-level socio-economic status [8] and comorbidities such as anxiety and depression [34] may have confounded the observed effect estimates. Fifthly, while the sample size of 107 permitted binary logistic regression analysis of factors associated with moderately common outcome events (i.e. adherence to dietary guidelines for fruits and whole red meats), this sample size gave relatively wide 95% CIs (i.e. greater uncertainty as to the true values of effect estimates) and was inadequate for analysing factors associated with less common or rare outcome events (i.e. adherence to dietary guidelines for vegetables, cereals/breads/grains and processed red meats) as well as one very common outcome event (i.e. adherence to the dietary guidelines for alcohol). Sixthly, the use of convenience sampling in one hospital setting has likely limited the generalisability of our results to other populations of rural cancer survivors.

Key strengths of the present study include the use of validated measures of food consumption and physical activity as well as the minimal missing data. The latter strength helped to circumvent selection bias and to maintain a reasonable sample size of over 100 for complete-case analysis of factors associated with moderately common outcome events. This is a particularly important consideration in rural health research given sampling frames are typically smaller than those available for metropolitan health research.

## Conclusion

The present study contributes preliminary data on levels of adherence to dietary guidelines and associated factors among rural Australian cancer survivors. Among 107 participants, levels of adherence to Australian dietary guidelines varied across food groups and were mostly low, albeit not markedly worse than Australia’s national population for the fruits and vegetables groups. While none of the assessed participant characteristics was associated with adherence to the dietary guideline for whole red meats, an age in the range 65–74 years and currently receiving cancer treatment were associated with greater and lower adherence, respectively, to the dietary guideline for fruits. There is a need for future studies to further investigate demographic, lifestyle-related, and health (including physical and mental health) factors associated with adherence to dietary guidelines among rural cancer survivors, ideally in larger samples and using longitudinal cohort study designs. Future larger studies conducted across different geographic locations could facilitate comparisons of dietary guideline adherence between rural and metropolitan cancer survivors, although researchers should be mindful of avoiding issues such as overpowered studies and any deficit reporting that is not constructive or not geographically contextualised. There is also a need for future studies in this area to consider tailored dietary recommendations for individuals with cancer, as opposed to generic guidelines, when determining adherence. While further research is needed, the present study’s findings nevertheless have implications for clinical practice. The low levels of adherence to dietary guidelines for most food groups in this study suggest a potential need to increase provision of dietary information, supportive care screening for dietary issues, and, wherever necessary, dietetics referrals, assessments, and interventions among rural cancer survivors. This approach to improving dietary quality among rural cancer survivors would require teamwork across a range of healthcare professionals, including oncologists, nurses, dieticians, and general practitioners, and could be refined through the development of tailored, localised models of supportive care based on future research and local rural contexts.

## Data Availability

The datasets that support the current study’s findings are not publicly available due to ethical/privacy restrictions. These datasets are, however, available from the corresponding author on reasonable request.
